# Good clinical outcomes, a high level of patient satisfaction and an acceptable re-operation rate are observed 7–10 years after augmented hip abductor tendon repair

**DOI:** 10.1007/s00167-023-07382-3

**Published:** 2023-03-20

**Authors:** Jay R. Ebert, Mikhil Jain, Gregory C. Janes

**Affiliations:** 1 School of Human Sciences (Exercise and Sport Science), University of Western Australia, 35 Stirling Highway, Crawley, Perth, WA 6009 Australia; 2 HFRC Rehabilitation Clinic, Perth, WA Australia; 3 Perth Orthopaedic and Sports Medicine Research Institute, Perth, WA Australia; 4 Leicester General Hospital, Leicester, England; 5 Perth Orthopaedic and Sports Medicine Centre, Perth, WA Australia

**Keywords:** Hip abductor tendon, Hip abductor tears, Surgical repair, Clinical outcomes

## Abstract

**Purpose:**

To investigate the clinical outcome, level of patient satisfaction, re-injury and re-operation rates of patients 7–10 years after augmented hip abductor tendon repair.

**Methods:**

Between October 2012 and May 2015, 146 patients were referred to the senior author with symptomatic hip abductor tendon tears, of which 110 (101 female, 92%) were included in the current study and underwent hip abductor tendon repair augmented with LARS. Patients had a mean age of 63.2 years (range 43–82), body mass index of 27.8 (range 20.0–40.2) and duration of symptoms of 3.6 years (range 6 months–18 years). Patient-reported outcome measures (PROMs) were evaluated pre-operatively and at 3, 6, 12 and 24 months, as well as 7–10 years post-operatively, including the Oxford Hip Score (OHS), 12-item Short Form Health Survey (SF-12), a Visual Analogue Pain Scale (VAS) evaluating the frequency (VAS-F) and severity (VAS-S) of hip pain, and patient satisfaction. Adverse events, surgical failures, revisions and subsequent treatments on the ipsilateral hip were reported.

**Results:**

A significant improvement (*p* < 0.05) was observed for all PROMs and, while a mean deterioration was observed for all PROMs from 24 months to final review (7–10 years), these were not significant (n.s.). In the 90 patients retained and assessed at final review, 93% were satisfied with their hip pain relief and 89% with their ability to participate in recreational activities. Overall, 9 (of 110, 8.2%) surgical failures were observed over the 7–10-year follow-up period.

**Conclusions:**

Good clinical scores, a high level of patient satisfaction and an acceptable re-injury rate were observed at 7–10 years after augmented hip abductor tendon repair, demonstrating satisfactory repair longevity.

**Level of evidence:**

IV.

## Introduction

Greater trochanteric pain syndrome (GTPS) affects up to 25% of the general population [25, 37]. While the exact incidence of hip abductor tendon tears in those presenting with symptomatic GTPS has not been reported to the best of our knowledge, their contribution is now acknowledged more widely and highlighted by the array of open, endoscopic and augmented surgical techniques now reported [2, 7, 11, 13, 15, 16, 18, 22, 23]. Published reviews have reported encouraging outcomes overall [1, 4, 8, 19–21, 26, 39], with patient satisfaction rates ranging from 66 to 90%, [3, 31, 32, 36] and similar clinical and functional outcomes in patients undergoing open and endoscopic hip abductor tendon repair methods [1, 4]. However, a higher complication and re-tear rate has been reported with open (versus endoscopic) repair [1, 26], with a review published by Alpaugh et al. [1] reporting an overall surgical complication rate of 13% in those undergoing open repair (with a 9% re-tear rate), versus a complication rate of 3% in those undergoing endoscopic repair (with no re-tears).

Furthermore, despite the growing evidence base and array of surgical hip abductor tendon repair options, limited studies report clinical outcomes in more than 30 patients [9, 11, 13, 14, 28, 30, 35, 38], and Kenanidis et al. [21] recently outlined the need for further high-quality studies with standardisation of pre-operative evaluation of patients and reporting of outcomes. In addition, while some studies have specifically reported a mid-term follow-up [5, 14, 28], published prospective (and retrospective) cohort studies rarely report outcomes beyond 2 years post-surgery. While this may provide a satisfactory timeframe to report short-term pain relief and recovery of function, it provides no information on the longevity of the surgical repair and longer-term patient satisfaction with the operation.

The current study sought to prospectively recruit and review a large series of patients now at 7–10 years following open hip abductor tendon repair augmented with a synthetic ligament, to ascertain the long-term clinical outcome and satisfaction level of patients, as well as repair longevity. The augmented surgical technique had been proposed to reduce the high re-tear rates reported in some studies, with encouraging patient outcomes up until 2 years previously reported for this technique [9, 11]. The current study hypothesised that: (1) a significant improvement in clinical outcomes would be observed over the 7–10-year post-operative period, with no significant decline in scores from 2-year to final 7–10-year review, (2) a surgical failure rate lower than that reported in prior research using an open repair surgical technique [1, 4] would be observed over the 7–10-year post-operative period, and (3) a high level (> 80%) of patient satisfaction would be observed in patients at final 7–10-year follow-up.

## Materials and methods

**Fig. 1:**
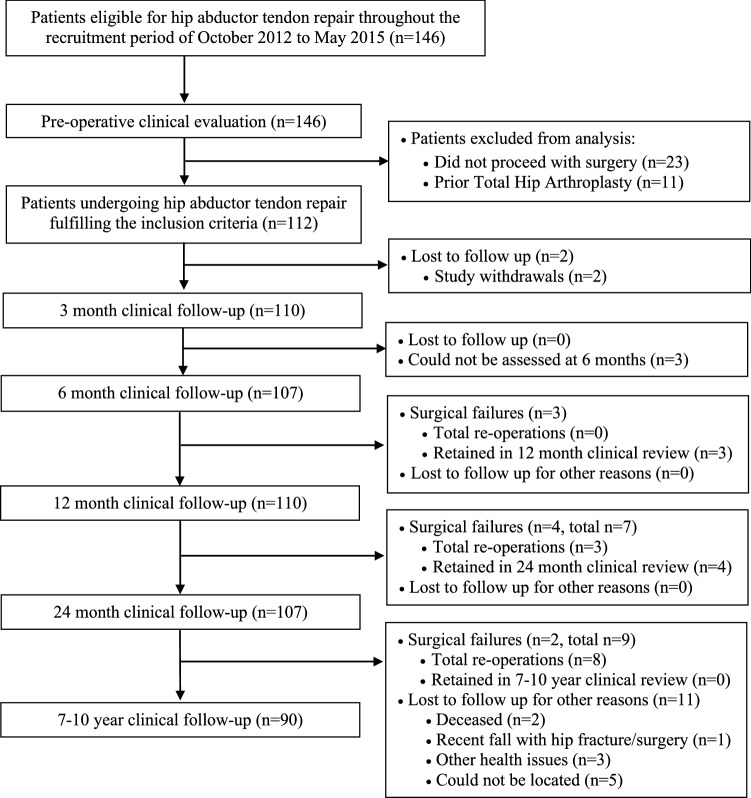
Study flowchart demonstrating patient recruitment and clinical evaluation

The written informed consent was obtained for all patients prior to study recruitment, while the Hollywood Private Hospital (HPH) Human Research Ethics Committee (HREC) approved the project (HPH348). As previously reported [11], 146 patients were referred to the senior author’s (GJ) orthopaedic practice between October 2012 and May 2015, of which 112 were included in the current prospective study, subsequently undergoing augmented hip abductor tendon repair (Fig. 1). The indications for the surgical treatment of hip abductor tendon tears in patients presenting with symptomatic GTPS through a standard clinical pathway in our institution include magnetic resonance imaging (MRI) diagnosed hip abductor tendon tearing and significant functional disability that has failed non-operative treatment (generally consisting of physiotherapy and injections) for a period of at least six months. Therefore, all patients included in the current study presented with symptomatic hip abductor tendon tears diagnosed via MRI, which included partial or full thickness tears of gluteus minimus in all cases, along with the anterior portion of gluteus medius. All patients had previously failed a course of non-operative treatment including corticosteroid injections and physiotherapy.

Of the 112 patients that were recruited into the current study and underwent surgery, two patients withdrew before the first 3-month clinical review due to reported time and travel restraints (Fig. 1). Therefore, the remaining 110 patients (101 female, 92%) presented with a mean age of 63.2 years (range 43–82), body mass index (BMI) of 27.8 (range 20.0–40.2) and duration of symptoms (DOS) of 3.6 years (range 6 months–18 years), while previously undergoing a mean of 3.3 (range 1–8) corticosteroid injections. Included in the study cohort and as previously reported [11], were eight patients with predominant symptoms of lateral-sided trochanteric pain with radiation down the lateral leg, though MRI-based evidence of advanced (Grade 2–4) [24] and/or symptomatic hip osteoarthritis (OA). Furthermore, two patients had previously undergone prior failed non-augmented hip abductor tendon repair, while two patients had previously undergone iliotibial band (ITB) release and/or bursectomy. Of the 110 patients assessed pre-operatively and retained after surgery, 110, 107, 110, 107 and 90 were assessed at 3 months, 6 months, 12 months, 24 months and 7–10 years post-operatively, respectively (Fig. 1).

### Augmented hip abductor tendon repair and post-operative management

**Fig. 2:**
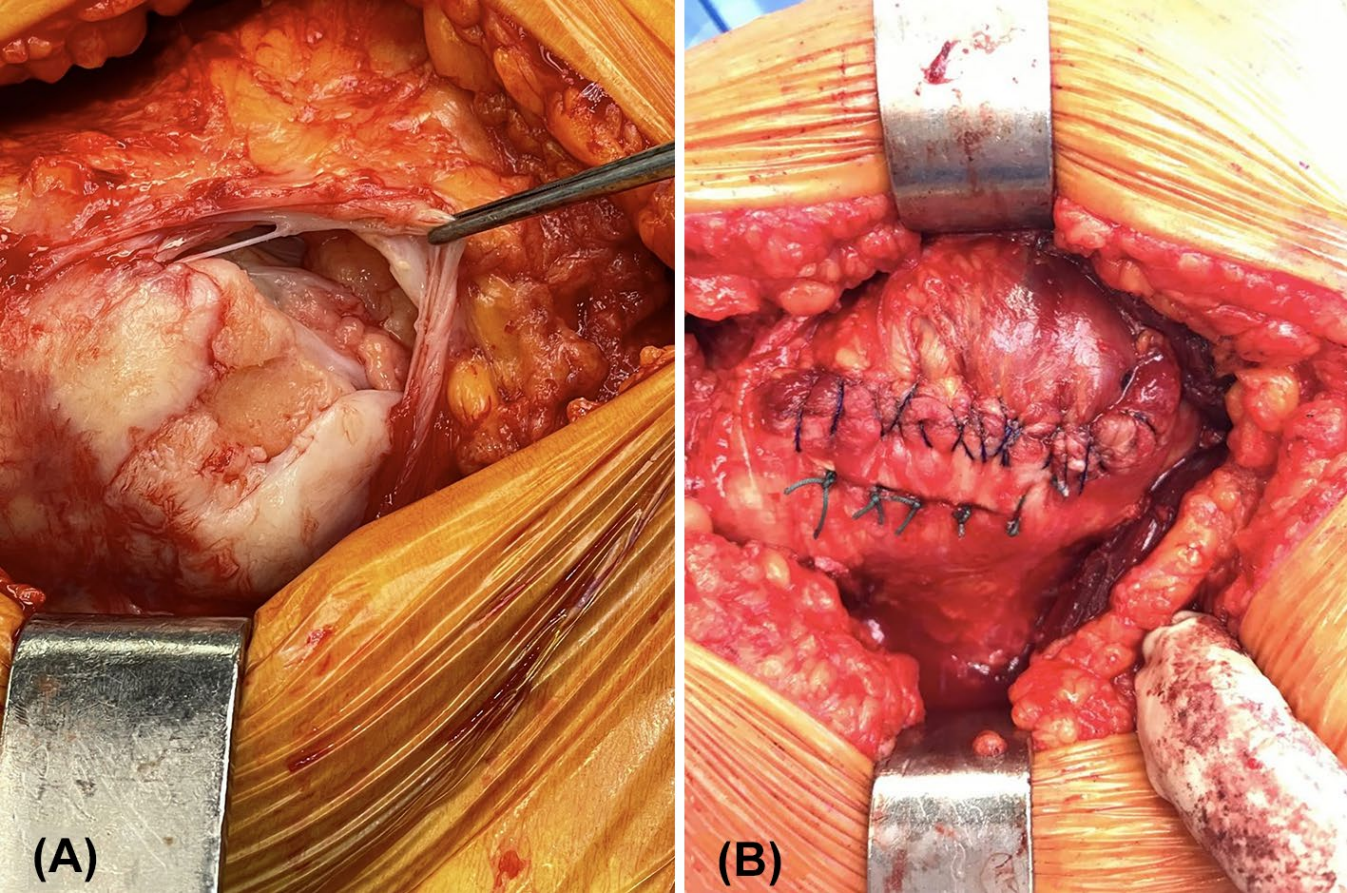
Intra-operative image demonstrating: **A** delamination of the hip abductor tendon tear pre-surgery, and **B** the final repair construct fixed to the greater trochanter with a series of interosseous sutures, with the LARS ligament not visible as it is on the deep surface of the hip abductor tendons

The surgical technique has been described in detail previously [9, 11]. Briefly, under general anaesthetic and via a direct lateral approach the trochanteric bursa was excised and the gluteus medius and minimus tendons were elevated from the trochanter, with the underlying bone subsequently decorticated to expose a bleeding bone surface. The delamination of the hip abductor tendon tear was first dealt with using transtendinous sutures and the broad end of a LARS ligament (ACTOR 10, Corin Group, Cirencester, UK) was sutured to the deep surface of the abductor tendon. Two converging bone tunnels were drilled, first from the gluteus minimus footprint on the anterior facet of the trochanter to midway through the trochanter, and second from postero-distal on the lateral prominence of the trochanter to meet the first tunnel. The LARS ligament was passed through the trochanter, subsequently fixed with an interference screw (Corin, Cirencester, UK). The tendon was fixed to the greater trochanter with a series of interosseous sutures and a bone anchor at the superior apex of the repair. Figure 2 demonstrates the pre-operative hip abductor tendon tear and the final repair construct. The rehabilitation programme has been previously described [9, 11, 12].

### Patient assessment

Patients were assessed with a range of patient-reported outcome measures (PROMs) pre-operatively and at 3, 6, 12 and 24 months, as well as 7–10 years post-surgery. First, the Oxford Hip Score (OHS) was employed, scored from 0 to 48 [6, 34], with a minimal clinically important difference (MCID) of 5.2 points previously reported after hip arthroplasty [40]. Second, the Mental (MCS) and Physical (PCS) Component Subscales of the 12-item Short Form Health Survey (SF-12), a Visual Analogue Pain Scale (VAS) evaluating the frequency (VAS-F) and severity (VAS-S) of hip pain on a scale of 0–10 (0 = no pain, 10 = constant/worst pain), and an 11-point Global Rating of Change (GRC) scale [17] evaluating the patient’s perceived status compared to before their surgery, ranging from − 5 (very much worse) to 0 (about the same) to 5 (completely recovered), were also employed. Finally, a patient satisfaction questionnaire was employed at final review (7–10 years post-surgery) to evaluate satisfaction with the surgery overall, as well as satisfaction with the surgery to relieve pain, improve the ability to perform activities of daily living (ADLs) and improve the ability to return to recreational activities (such as swimming, cycling, dancing, golf). A Likert response scale was employed with descriptors: very satisfied, somewhat satisfied, somewhat dissatisfied and very dissatisfied.

### Statistical analysis

Prior to study onset and as reported previously [11], a priori sample size was calculated based on improvement in the OHS over time. An earlier study undertaken in a similar pilot augmented hip abductor tendon repair cohort had reported that a sample size of 22 patients would have over 99% power to detect a mean change of five points in the OHS [3], which had been suggested as the minimal clinically important difference for the OHS [34], assuming a standard deviation of the change score of 10, corresponding to a moderate effect size of 0.5. Given the early success observed with the surgical technique and steady flow of patients requiring and undergoing the surgical procedure, study recruitment was significantly increased and continued.

Means (SD and range) were calculated and presented for all PROMs. Analysis of variance (ANOVA) was employed to investigate the pre- and post-operative change in PROMs (OHS, VAS-F, VAS-S, SF-12 PCS and MCS) over time, as well as evaluate the post-operative change in the GRC scale. T-tests were also employed to specifically evaluate any change from 24 months post-surgery to final post-operative review (7–10 years). The number (and percentage) of patients reporting ‘very satisfied’, ‘Somewhat Satisfied’, ‘Somewhat Dissatisfied’ and ‘Very Dissatisfied’ within each of the satisfaction domains at 7–10 years (final follow-up) was presented. The number (and type) of surgical complications, early post-operative adverse events, surgical failures and revisions were presented, as was an overview of any subsequent treatments (injections) or other surgical procedures the patient had required on the ipsilateral hip since the primary hip abductor tendon repair. Where appropriate, statistical analysis was performed using SPSS software (SPSS, Version 27.0, SPSS Inc., USA), with statistical significance determined at *p* < 0.05.

## Results

### Clinical scores

**Fig. 3:**
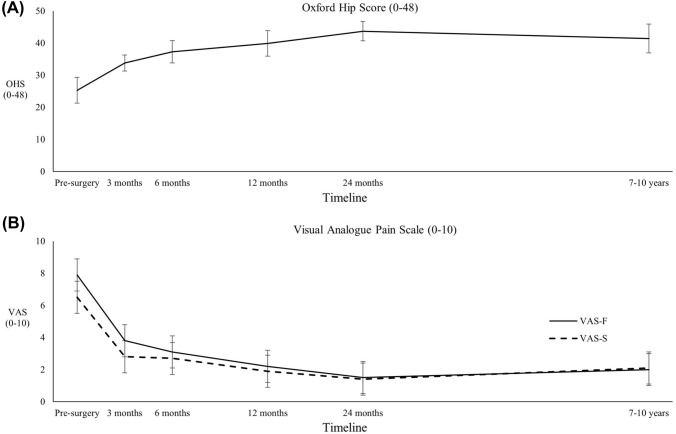
Patient-reported outcomes demonstrating significant improvement over the pre- and post-operative timeline, without significant deterioration from 24-month to 7–10-year follow-up, including: **A** the Oxford Hip Score (OHS), and **B** the Visual Analogue Pain Scale (VAS), including the Frequency (VAS-F) and Severity (VAS-S) of hip pain

A significant improvement (*p* < 0.05) was observed for all PROMs over the evaluation period (Table 1). While a mean deterioration was observed for all PROMs from 24 months to final review (7–10 years), this was not statistically significant (n.s.) for every PROM including the OHS, the VAS-F and VAS-S (Fig. 3). In the 90 patients retained and assessed at final review, 84 (93.4%) were satisfied with the surgery to relieve their hip pain, 82 (91.1%) with the improvement in their ability to undertake ADLs, 80 (88.9%) with their ability to participate in recreational activities, and 82 (91.1%) were satisfied overall (Table 2).

**Table 1:** Patient-reported outcome measures (PROMs) over the pre- and post-operative period to final review (7–10 years post-surgery), including the Oxford Hip Score (OHS), the Mental (MCS) and Physical (PCS) Component Subscales of the 12-item Short Form Health Survey (SF-12), the frequency (VAS-F) and severity (VAS-S) of hip pain, and the Global Rating of Change (GRC) scale

Variable	Pre-surgery	3 months	6 months	12 months	24 months	7–10 years	*p* value
OHS							
Mean (SD)	25.3 (8.7)	33.8 (8.4)	37.3 (7.9)	39.9 (6.7)	43.7 (5.4)	41.4 (7.9)	< 0.0001
Range	5–46	12–47	11–48	23–48	22–48	13–48	
SF-12 (PCS)							
Mean (SD)	33.2 (9.2)	36.4 (10.5)	40.6 (10.1)	44.1 (9.4)	46.2 (9.1)	42.2 (9.3)	< 0.0001
Range	9.0–57.8	10.8–57.2	10.8–61.4	14.9–58.1	23.5–58.1	20.2–57.6	
SF-12 (MCS)							
Mean (SD)	49.3 (11.5)	53.5 (11.4)	52.4 (11.4)	54.9 (9.8)	55.5 (8.8)	51.9 (9.8)	0.020
Range	20.4–70.8	25.6–70.9	27.3–69.7	27.3–69.5	23.0–69.5	24.9–67.1	
VAS-F							
Mean (SD)	7.9 (2.6)	3.8 (2.8)	3.1 (2.6)	2.2 (2.0)	1.5 (1.9)	2.0 (2.1)	< 0.0001
Range	1–10	0–10	0–10	0–6	0–9	0–8	
VAS-S							
Mean (SD)	6.5 (2.2)	2.8 (1.8)	2.7 (2.1)	1.9 (1.5)	1.4 (1.5)	2.1 (2.2)	< 0.0001
Range	1–10	0–7	0–9	0–5	0–7	0–8	
GRC							
Mean (SD)	N/A	2.4 (1.9)	2.8 (1.8)	3.4 (1.7)	3.8 (1.4)	3.7 (1.7)	< 0.0001
Range	N/A	− 5 to 5	− 3 to 5	− 5 to 5	− 5 to 5	− 5 to 5

**Table 2:** Satisfaction scores for the 90 patients assessed at final review (7–10 years post-surgery), including the number (and percentage) of patients within each of the four satisfaction grading (very satisfied, somewhat satisfied, somewhat dissatisfied, very dissatisfied) for each of the four satisfaction items

Satisfaction item	Pain relief	Improving ability to undertake ADLs	Improving ability to participate in recreational activities	Overall satisfaction
Very satisfied	70 (77.8%)	69 (76.7%)	52 (57.8%)	67 (74.4%)
Satisfied	14 (15.6%)	13 (14.4%)	28 (31.1%)	15 (16.7%)
Dissatisfied	5 (5.5%)	7 (7.8%)	9 (10.0%)	7 (7.8%)
Very dissatisfied	1 (1.1%)	1 (1.1%)	1 (1.1%)	1 (1.1%)
Satisfied, *n* (%)	84 (93.4%)	82 (91.1%)	80 (88.9%)	82 (91.1%)

### Surgical complications, adverse events, failures/revisions and subsequent treatments

An overview of all surgical complications and early post-operative adverse events, surgical failures and revision procedures, and any other subsequent treatments or surgical procedures on the ipsilateral hip are outlined in Table 3. Overall, 9 (of 110, 8.2%) surgical failures were observed over the 7–10-year follow-up period, all of which were not included in the 7–10-year clinical follow-up (although only eight of these patients had since undergone revision hip abductor tendon repair). Of these, three failures were observed between 6 and 12 months, four failures between 12 and 24 months and a final two failures that were observed beyond 24 months post-surgery. Therefore, an overall re-tear rate over the 7–10-year period of 8.2% was observed. Of the nine surgical failures (of which five appeared to have occurred following secondary accidents and four for unknown reasons), eight of these had since been surgically revised which generally included removal of the LARS and revision repair with multiple interosseous sutures, though one patient underwent gluteus maximus transfer. Of further note, at the time of revision surgeries the proximal end of the LARS was still intimately attached to the under surface of the gluteal tendons and had to be dissected off for LARS removal. While non-specific inflammation with moderate fluid present and synovitis within the bursa was generally observed, no evidence of adverse reaction and/or particulate debris from the LARS that may be associated with abrasion or rupture was observed.

**Table 3:** Review of surgical complications and early post-operative adverse events, surgical hip abductor tendon repair failures and revision surgeries, and any other secondary hip surgical procedures or further hip-related treatments since the primary hip abductor tendon repair surgery

Surgical complications and early post-operative adverse events
Superficial wound infections (*n* = 3)
Haematoma (*n* = 1)
Deep vein thrombosis and development a pulmonary embolism (*n* = 1)
All reported complications were treated accordingly without any further issues
Surgical failures and revisions
Failures encountered within the first 12 months (*n* = 3)—confirmed via repeat MRI at 7, 9 and 11 months post-surgery
Failures encountered between 12 and 24 months (*n* = 4)—confirmed via repeat MRI at 13, 14, 14 and 16 months post-surgery
Failures encountered between 24-month and final 7–10-year follow-up (*n* = 2)—confirmed via repeat MRI at 4 and 6 years post-surgery
Reported reasons for recurrence of symptoms (and subsequently confirmed re-tear)—fall (*n* = 3), motor vehicle accident (*n* = 1), accident at the beach (*n* = 1), unknown (*n* = 4)
At the time of 7–10-year follow-up, 8 (of 9) observed surgical failures had undergone revision surgery (1 patient had not progressed towards revision surgery, though she had undergone augmented hip abductor tendon repair in the current study following failed hip abductor tendon repair previously via a non-augmented approach)
Further treatments and/or surgical procedures on ipsilateral hip
Corticosteroid injection into bursa (*n* = 3) or into hip joint (*n* = 1)
Total hip arthroplasty (*n* = 3)—at 6, 6 and 7 years following primary hip abductor tendon repair (all patients retained in 7–10-year review)
Other (*n* = 2)
One patient underwent proximal hamstring tendon repair following a secondary incident 5 years after her primary hip abductor tendon repair
One patient had a fall and sustained fractured neck of femur with subsequent fixation
Apart from the patient who had the fall and fractured her neck of femur, all other patients were retained in the 7–10-year review

Early surgical complications and/or adverse events for this cohort have been previously reported and included three superficial wound infections, one haematoma and 1 DVT (with development a pulmonary embolism) that were all treated accordingly without further issue [11]. Subsequent treatments (other than revision hip abductor tendon repair, including injections and other surgical procedures) that patients had required on the ipsilateral hip included four injections (three into the bursa and one into the joint, across four different patients), three hip replacements, one patient who required a proximal hamstring repair following a secondary rupture and one patient that had fallen and fractured her neck of femur requiring subsequent fixation, only weeks before her designated final review.

## Discussion

The most important finding from the current study was that a significant improvement in clinical scores and a high level of patient satisfaction was observed in patients undergoing augmented hip abductor tendon repair up until 2 years post-surgery, which was sustained out to 7–10 years. Furthermore, while this study reports on one of the largest and most thoroughly followed prospective hip abductor tendon patient cohorts, even at 7–10 years post-surgery the re-tear rate was lower than that reported in many other studies presenting outcomes after open hip abductor tendon repair, demonstrating sound longevity of the procedure.

The current study demonstrated a significant improvement over time in all PROMs employed, including the OHS which is one of the most commonly reported PROMs in patients undergoing hip abductor tendon repair [10], as well as the SF-12, VAS and the patient-perceived GRC scale. While most of the improvement across all PROMs occurred within the first 12 months and this has been previously reported [11], in an extended patient cohort it had been reported that the VAS and GRC may improve further between 12 and 24 months [9]. A minor (though non-statistical) decline in all PROMS was observed between the 2-year and final 7–10-year review, which may be due to a range of variables including age and/or other comorbidities, as well as undiagnosed progression of hip osteoarthritis or other hip pathologies. However, despite this small decline in all PROMs, this was not statistically significant across any PROM, with the improvement observed from pre-surgery to 7–10 years in the OHS still three times greater than the previously reported MCID [40]. This is in support of the first study hypothesis. Also worth noting, and when considering the (non-significant) decline in PROMs from 24 months to 7–10 years, is the fact that the included cohort had a mean age of 63 years (range 43–82) at surgery which was well beyond 70 years at final review. This also included 17 patients that were ≥ 75 years of age at the time of their operation, of which 15 were still retained in the final 7–10-year review, so a decline in some form was largely expected.

Patient satisfaction rates ranging from 66 to 90% have been reported after hip abductor tendon repair [3, 5, 27, 31–33, 36], with 96% of this cohort satisfied with their hip abductor tendon repair for relieving pain, 90% with their ability to return to recreational activities and 96% satisfied overall, at 12 months post-surgery [11]. In the 90 patients available for review at 7–10 years post-surgery as part of the current study, satisfaction rates of 93% (pain relief), 89% (participation in recreational activities) and 91% (overall satisfaction) were observed, again in support of the second hypothesis and certainly at the higher end of previously reported satisfaction rates, even at 7–10 years after surgery. These high satisfaction rates are supported by the mean GRC scales observed, which were 3.4, 3.8 and 3.7 at 12 months, 24 months and 7–10 years, respectively.

A number of re-tears were observed in the current study, reflective of an increase in post-operative symptoms that failed to settle, or a persistent increase in symptoms that extended well beyond the expected post-operative timeline, though all confirmed by subsequent MRI. An overall re-tear rate over the 7–10-year period of 8.2% was observed, which also supported the final hypothesis. As part of the current long-term review, we also sought to undertake a detailed review of subsequent treatments (other than revision hip abductor tendon repair, including injections and other surgical procedures) that patients had required on the ipsilateral hip. As reported, it was encouraging to see that few subsequent treatments were required in this cohort.

Some limitations are acknowledged in this study. While this appears one of the largest hip abductor tendon repair cohorts reported and with long-term review, it was a prospective study with no comparative arm (i.e. comparing the augmented hip abductor tendon repair to patients not undergoing surgery, or to a different or non-augmented technique). Furthermore, given the size of the cohort the assessment focus was on PROMs and satisfaction, together with re-tears, rather than a series of additional objective assessments that may have provided further information on patient strength and function, though may have proved more challenging to retain as many of the initially recruited cohort. Finally, the association between gluteal muscle fatty degeneration, atrophy and/or tear morphology on pre-operative MRI and post-operative outcome was not assessed which may affect clinical outcome [29]. Furthermore, post-operative MRI was not undertaken in the current study to assess the quality of the surgical repair and/or fatty atrophy of the gluteal muscles, unless indicated clinically due to concerns over the failure of the repair as outlined above. As the clinical relevance, this study highlights the adjunctive role of synthetic augmentation of primary hip abductor tendon repair, as a means of providing significantly improved and sustained long-term clinical outcomes, with a high level of reported patient satisfaction and a relatively low re-tear rate.

## Conclusion

The current study has demonstrated that hip abductor tendon repair, augmented with a synthetic ligament, results in significantly improved and sustained long-term clinical outcomes, with a high level of reported patient satisfaction and a relatively low re-tear (and re-operation) rate, with most patients not requiring any further ipsilateral hip-related treatment beyond their primary hip abductor tendon repair.

## Data Availability

The data collected and analyzed for the current study are available from the corresponding author on reasonable request.
